# Identification of Disease-Related 2-Oxoglutarate/Fe (II)-Dependent Oxygenase Based on Reduced Amino Acid Cluster Strategy

**DOI:** 10.3389/fcell.2021.707938

**Published:** 2021-07-16

**Authors:** Jian Zhou, Suling Bo, Hao Wang, Lei Zheng, Pengfei Liang, Yongchun Zuo

**Affiliations:** ^1^State Key Laboratory of Reproductive Regulation and Breeding of Grassland Livestock, College of Life Sciences, Inner Mongolia University, Hohhot, China; ^2^College of Computer and Information, Inner Mongolia Medical University, Hohhot, China

**Keywords:** 2-oxoglutarate/Fe (II)-dependent oxygenase, reduced amino acid cluster, machine learning, anova, incremental feature selection, 10-fold cross-validation test

## Abstract

The 2-oxoglutarate/Fe (II)-dependent (2OG) oxygenase superfamily is mainly responsible for protein modification, nucleic acid repair and/or modification, and fatty acid metabolism and plays important roles in cancer, cardiovascular disease, and other diseases. They are likely to become new targets for the treatment of cancer and other diseases, so the accurate identification of 2OG oxygenases is of great significance. Many computational methods have been proposed to predict functional proteins to compensate for the time-consuming and expensive experimental identification. However, machine learning has not been applied to the study of 2OG oxygenases. In this study, we developed OGFE_RAAC, a prediction model to identify whether a protein is a 2OG oxygenase. To improve the performance of OGFE_RAAC, 673 amino acid reduction alphabets were used to determine the optimal feature representation scheme by recoding the protein sequence. The 10-fold cross-validation test showed that the accuracy of the model in identifying 2OG oxygenases is 91.04%. Besides, the independent dataset results also proved that the model has excellent generalization and robustness. It is expected to become an effective tool for the identification of 2OG oxygenases. With further research, we have also found that the function of 2OG oxygenases may be related to their polarity and hydrophobicity, which will help the follow-up study on the catalytic mechanism of 2OG oxygenases and the way they interact with the substrate. Based on the model we built, a user-friendly web server was established and can be friendly accessed at http://bioinfor.imu.edu.cn/ogferaac.

## Introduction

**FIGURE 1:**
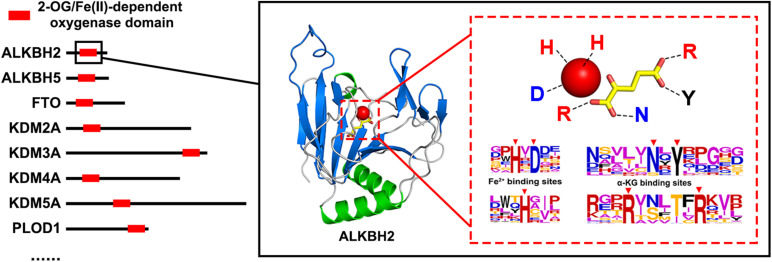
Schematic diagram of the structure of 2-oxoglutarate/Fe (II)-dependent (2OG) oxygenase.

2-Oxoglutarate/Fe (II)-dependent (2OG) oxygenases (EC:1.14.11), generally using nonheme iron as an active-site cofactor, promote oxidative decarboxylation of the substrate to produce carbon dioxide and succinic acid (Hausinger, 2004; Hewitson et al., 2005; Islam et al., 2018). 2OG oxygenases, which can catalyze many different oxidation reactions, are a superfamily with members widely distributed in animals, plants, and microorganisms. In animals, their catalytic range includes hydroxylation and N-demethylation proceeding *via* hydroxylation; in plants and microbes, they affect a wider range, including hydroxylation, ring formations, cleavage, oxidation, rearrangements, desaturations, and halogenations (Farrow and Facchini, 2014; Kawai et al., 2014). The proteins of this superfamily can be divided into 2OG oxygenase domain-containing oxygenases and JmjC domain-containing oxygenases (Jia et al., 2017). Figure 1 is a schematic diagram of the structure of 2OG oxygenases.

Due to the diversity of 2OG oxygenases and the wide range of binding substrates, these oxygenases play an important role in physiology and have high therapeutic value and therapeutic potential as targets in cancer and many other diseases (Rose et al., 2011). For example, the protein containing the JmjC domain (JMJD6) is located in the nucleus that catalyzes lysine hydroxylation and arginine demethylation of histone and non-histone peptides (Chang et al., 2007; Liu et al., 2013). JMJD6 promotes cell proliferation and migration *in vitro* and accelerates tumor growth *in vivo*, so it may become an attractive target for a new generation of anticancer drugs (Lin et al., 2006; Lee et al., 2012). Prolyl 4-hydroxylase (P4H) plays a vital role in the synthesis of collagen and the regulation of oxygen homeostasis. Collagen P4Hs are considered to be attractive targets for drug inhibitors and involved in the treatment of fibrotic diseases and cancer metastasis (Vasta and Raines, 2018). Hypoxia-inducible transcription factor-prolyl 4-hydroxylase inhibitors are believed to have beneficial effects in the treatment of diseases such as myocardial infarction, stroke, peripheral vascular disease, diabetes, and severe anemias (Myllyharju, 2008; Liao and Zhang, 2020). ALKB homologs (ALKBH) homologs can regulate the physiological and pathological processes of cardiovascular diseases (CVDs), which have great potential in the development of CVD drugs and are expected to become a potential target for the treatment of CVD (Xiao et al., 2020). The change in the catalytic activity or expression level of lysine demethylases (KDMs) is closely related to many diseases, including cancer genesis and progression, neurological disorders, inflammatory and immune disorders, metabolic diseases, and regenerative diseases. Modulators/inhibitors of KDMs may be used as new treatments for cancer and other diseases (Arifuzzaman et al., 2020). Therefore, it is particularly meaningful to predict 2OG oxygenases and find more potential 2OG oxygenases. Since the identification of 2OG oxygenase is time-consuming and expensive, machine learning is an effective and fast method to predict it.

In the past, many machine learning methods for the prediction of metal ion-binding proteins have achieved excellent results. For example, Lin et al. (2006) applied the sequence information used by support vector machine (SVM) to predict the metal ion-binding protein and got a relatively marvelous prediction result. Mohan et al. (2010) used a set of physicochemical parameters of metal ion-binding proteins encoded by the three genes *CzcA*, *CzcB*, and *CzcD* as the training set of the supervised classifier, establishing a model to identify metal ion-binding proteins from unknown proteins. Valasatava et al. (2016) developed MetalPredator, a web server used to predict iron–sulfur cluster-binding proteomes, and it featured an excellent performance in terms of precision and recall. Many studies have also achieved good results in the prediction of metal ion-binding sites, including iron ion-binding sites (Liu and Hu, 2011; Liou et al., 2014), zinc ion-binding sites (Shu et al., 2008; Chen et al., 2013; Yan et al., 2019), copper ion binding sites (Levy et al., 2009; Brylinski and Skolnick, 2011). The above indicate that machine learning is suitable for the application of metal ion-binding proteins (Valasatava et al., 2016). Not only that, studies have shown that using the reduced amino acid cluster (RAAC) strategy to predict the types of proteins can reduce noise and achieve higher accuracy (Zheng et al., 2019). In the prediction of human and nonhuman enzymes (Wang H. et al., 2021), ion channel-targeted conotoxins (Sun et al., 2020), plasmodium secretory protein (Zhang et al., 2020), and defensin peptides (Zuo et al., 2019), the method of reduced amino acid has shown superior performance.

**FIGURE 2:**
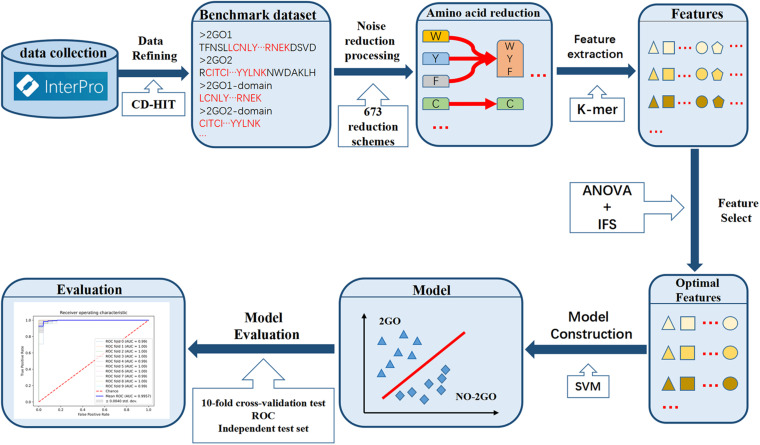
The workflow of OGFE_RAAC predictor.

In this study, we established a prediction model for 2OG oxygenases based on SVM, which can effectively identify 2OG oxygenases. A new feature representation scheme (amino acid reduction cluster) was involved in this work. The RAAC strategy can greatly decrease the complexity of protein sequences and extremely reduce the use of computer memory (Zuo et al., 2017; Zheng et al., 2019). The workflow of constructing the OGFE_RAAC is shown in Figure 2. Firstly, an objective dataset was established, which contains 734 2OG oxygenases and 385,381 non-2OG oxygenases from the InterPro database. Subsequently, reduced amino acid composition combined with K-mer strategy was used to represent sequence features, and the optimal one was selected from 673 reduction schemes (Zuo et al., 2015). At the same time, we obtained the best feature combination through analysis of variance (ANOVA) combined with incremental feature selection (IFS) and applied SVM to establish the model. The results of 10-fold cross-validation and independent test set showed that OGFE_RAAC could accurately predict 2OG oxygenases.

## Materials and Methods

### Dataset

The 2OG oxygenase superfamily can be classified into 2OG oxygenase domain-containing oxygenases and JmjC domain-containing oxygenases, so we collected all the verified 734 proteins of these two domains in the IPR number (IPR005123 and IPR003347) of the InterPro public database as a positive sample. Concurrently, 385381 protein data verified by SwissProt were gathered as negative samples, which is the manual annotation and review part of UniProt. Then, CD-HIT (Huang et al., 2010) was used to remove sequences with a similarity of more than 50% (Zou et al., 2020), and 480 samples are selected as the training set (Fu et al., 2012). We chose 150 samples from the rest as the test set, and the dataset was named 2OG-SwissProt. For the purpose of getting a better model, we also used iron-binding protein as a negative sample to construct a dataset. We acquired 593 iron-binding proteins (GO:0005506, 2OG oxygenase proteins removed) from the InterPro public database and processed them in the same way as the 2OG-SwissProt dataset to obtain 471 training set samples and 159 test set samples; the dataset was named 2OG-Fe.

For further research, we manually extracted the domain sequences of 2OG oxygenase and iron-binding proteins. The processing method is the same as the above; in order to better verify the prediction results, we used CD-HIT processing sequence similarity less than 50% as the training set and the rest as the independent test set. Among them, 1,036 samples constitute an independent test set, 621 positive samples and 415 negative samples; 283 samples constitute a training set, 113 positive samples and 170 negative samples. This dataset was named 2OG-domain (Table 1).

**TABLE 1:** Data composition of each dataset.

Dataset	Group	Training set	Test set
2OG-SwissProt	Positive	240	75
	Negative	240	75
2OG-Fe	Positive	240	75
	Negative	231	84
2OG-domain	Positive	113	621
	Negative	170	415

### Reduce Protein Sequence

Under normal circumstances, protein is composed of 20 natural amino acids. We combine amino acids with similar characteristics based on the physicochemical properties and atomic arrangement of amino acids. For instance, using fuzzy clustering technology and matrices cluster amino acids and interpret the sequence in a new encoding method (Georgiou et al., 2009; Zuo and Li, 2009). The strategy of RAACs can effectively reduce the complexity of the sequence and improve computational efficiency. In the study, we used 673 amino acid reduction schemes generated by 74 types to predict 2OG oxygenases, and each type has a reduced size of 2–19 (Zuo et al., 2019; Zheng et al., 2020).

### Extract Features Based on K-mer

The typical K-mer (N-peptide) composition can effectively dig out the detailed information of the amino acid composition of the sequence (Zhu et al., 2019; Jaillard et al., 2020). We use K-mer (*K* = 1, 2, 3) to extract amino acid sequence information. Due to the limited memory, the maximum *K* value is 3, and a total of 20*^*K*^* features can be obtained according to the original amino acid composition. The composition of K-mer (*K* = 2) can be expressed as follows:

(1) $$P=R_{1}\,R_{2}\,R_{3}\,\cdots \,R_{L-1}\,R_{L}$$

(2) $$F={\left[d_{1},d_{2},\cdots \,d_{400}\right]}^{T}$$

Here, *R*_*i*_ represents the *i*-th residue of the 2OG oxygenases.*L* represents the total length of the amino acid sequence. *d*_*i*_ (*i* = 1, 2,…, 400) is the *i*-th dipeptide in the 400-amino acid combination, and T means transposition operator. The *d*_*i*_ can be calculated as follows:

(3) di=ni∑i=1400ni

Here, *n*_*i*_ denotes the number of the *i*-th dipeptide. Combined with RAAC strategy, the feature extraction method can be expressed as follows:

(4) $$F=\,\left[P_{1,1}^{1},P_{1,2}^{2},\ldots ,P_{i,j}^{k},\ldots ,P_{T,C}^{N}\right]$$

where $P_{i,j}^{k}$ denotes the method of the N-peptide with different RAAC descriptors (N-peptide). *N* denotes the N-peptide. *T* denotes the type of different amino acid alphabets, and *C* denotes the cluster of the reduced amino acid alphabet. The parameters of the above equation can be limited as follows:

(5) $$\{\begin{array}{c} 1\leq k\leq N,N=\left[1,2,3\right]\\ 1\leq i\leq T,T=\left[1,2,\ldots ,74\right]\\ 1\leq j\leq C,C=\left[2,3,\ldots ,19\right] \end{array}$$

### Support Vector Machine

Support vector machine is a machine learning model that classifies data according to supervised learning methods and has been widely used in bioinformatics (Beer, 2017; Huang et al., 2018; Manavalan et al., 2018; Meng et al., 2020; Tahir and Idris, 2020). There are four types of kernel function, including linear functions, polynomial functions, S-shaped functions, and radial basis functions (RBFs). In the past predictions of proteins, the RBF kernel function had better performance, and we have verified that the RBF kernel function has better performance in our model through the calculation and comparison of the four kernel functions. Accordingly, we used the SVM package with RBF kernel for the classifier, which can be obtained from https://www.csie.ntu.edu.tw/~cjlin/libsvm (Chang and Lin, 2011). The libsvm package provides a grid search program to optimize the parameters *C* and γ. The kernel parameter γ and the regularization parameter *C* are used to adjust the SVM model to obtain the best performance. The selection ranges of *C* and γ are as follows:

(6) $$2^{-5}<C<2^{15}$$

(7) $$2^{-15}<\gamma <2^{3}$$

### Feature Screening

The initial features extracted by K-mer are exclusive features, not the optimal combination of features (Zou et al., 2016; He et al., 2020). ANOVA is a popular feature selection method that can help us measure the weight value of each feature (Saeys et al., 2007; Tang et al., 2018). Then, we used IFS to determine the dimensionality of the best feature set according to the feature weights obtained by the ANOVA. The ANOVA equations are as follows:

(8) $$F=\frac{S_{x}^{2}}{S_{y}^{2}}$$

(9) $$S_{x}^{2}=\frac{1}{n-1}\,\sum_{i=1}^{n}{\left(x_{i}-\bar{x}\right)}^{2}$$

(10) $$S_{y}^{2}=\frac{1}{m-1}\,\sum_{i=1}^{m}{\left(y_{i}-\bar{y}\right)}^{2}$$

where *F* is the variance value of the feature. $S_{x}^{2}$ is the sample variance between groups. $S_{y}^{2}$ denotes the sample variance within groups.

### Performance Evaluation

In statistical prediction, the following three cross-validation methods are often used to examine a predictor for its effectiveness in practical application: independent dataset test, subsampling (K-fold cross-validation) test, and jackknife test. However, among the three cross-validation methods, the jackknife test is deemed the least arbitrary that can always yield a unique result for a given benchmark dataset and hence has been increasingly used and widely recognized by investigators to examine the accuracy of various predictors (Chou and Shen, 2008; Chou, 2011; Chou et al., 2012; Zhang et al., 2021). However, since the current study would involve feature selection as described above, to reduce the computational time, the 10-fold cross-validation test and independent dataset test would be adopted as done by many investigators using SVM as the prediction engine. The performance can be measured in term of Sensitivity (Sn), Specificity (Sp), F1 score, Matthew’s correlation coefficient (MCC), and Accuracy (Acc; Li et al., 2020; Shen and Zou, 2020; Yang et al., 2021), which are expressed as follows:

(11) $$\text{Sn}=\frac{T\,P}{T\,P+F\,N}$$

(12) $$\text{Sp}=\frac{T\,N}{T\,N+F\,P}$$

(13) $$F\,1\,\text{score}=\frac{2\,T\,P}{2\,T\,P+F\,P+F\,N}$$

(14) $$\text{Acc}=\frac{T\,P+T\,N}{T\,P+F\,N+T\,N+F\,P}$$

(15) $$\text{MCC}=\frac{T\,P\times T\,N-F\,P\times F\,N}{\sqrt{\left(T\,P+F\,P\right)\,\left(T\,N+F\,N\right)\,\left(T\,P+F\,N\right)\,\left(T\,N+F\,P\right)}}$$

where *TP*, *TN*, *FP*, and *FN* represent true-positive, true-negative, false-positive, and false-negative samples, respectively.

## Results

### Predictive Performance of Different Reducing Amino Acid Cluster

**FIGURE 3:**
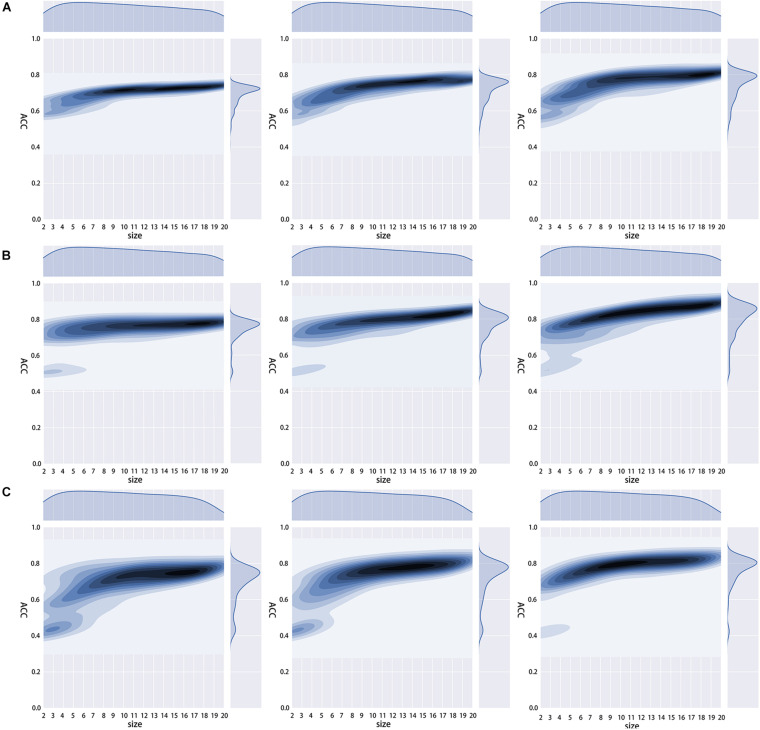
Density distribution diagram of different *K* value accuracy rates. **(A–C)** are the density distribution diagrams of the 2OG-SwissProt set, 2OG-Fe set, and 2OG-domain set at different *K* values in 673 reduction schemes.

To obtain the optimal amino acid reduction scheme and the appropriate *K* value (*K* = 1, 2, 3), we calculated the accuracy of the 673 reduction schemes mentioned in RAACBook (Zheng et al., 2019) with the different *K* values. We found that all three models showed the best performance at *K* = 3, and most of the reduction schemes had higher accuracy when *K* = 3 (Figure 3). We guessed that there would be more features when *K* = 3, and they would better reflect the properties of the protein and get a more accurate model.

**FIGURE 4:**
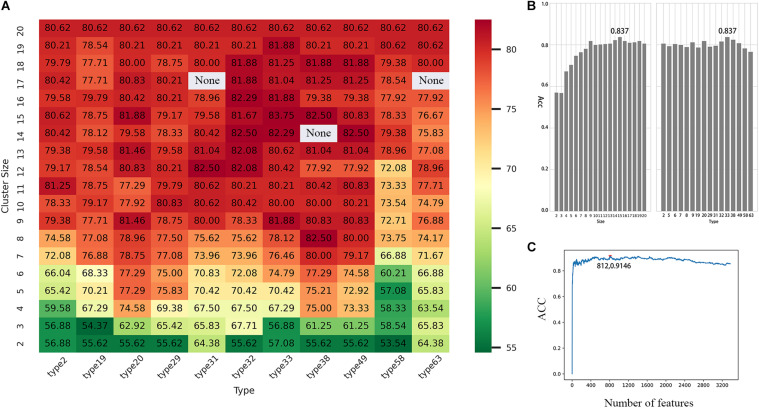
Performance evaluation of different reduced amino acid clusters. **(A)** Heat map of accuracy distribution of different reduced amino acid clusters. **(B)** The accuracy rate of the reduced amino acid cluster (*t* = 33, *s* = 15) with the highest accuracy rate reaches 83.75%. **(C)** The incremental feature selection (IFS) curve shows that prediction accuracy is 91.46% when using 812 optimal features based on the tripeptide combination (*t* = 33, *s* = 15).

After confirming that the model has better performance when *K* = 3, we then selected the best scheme from 673 RAAC schemes to construct the model. In the 2OG-SwissProt model, we tested each size of each reduction type and compared different reduction sizes of different reduction types (Figure 4A). We found that when *t* = 33 (Table 2), *s* = 15 (*t* represents the *t*-th reduction type in RAACBook; *s* represents the size of the RAAC), the highest accuracy rate is 83.75% (Figure 4B). In the prediction of the 2OG-Fe dataset, we were pleasantly surprised to find that the highest accuracy rate also appears in the reduction type 33, and the highest accuracy rate is 90.04% when *s* = 16 (Supplementary Figure 1B). There is also a very high accuracy rate at *s* = 15, reaching 88.76% (Supplementary Figure 1A). The reduction method of type 33 uses a database of aligned protein structures to propose a new clustering method based on the substitution scores, which aggregates 20 amino acids in two groups, namely, the hydrophobic groups and the polar groups (Li and Wang, 2007). Therefore, we speculated that the function of 2OG oxygenases may be related to its polarity and hydrophobicity.

**TABLE 2:** Cluster size of reduced amino acid alphabet of type 33.

Size	Reduced amino acid cluster
2	STANDGRQEKHPIVLMWYF-C
3	STANDGRQEKHP-IVLMWYF-C
4	STANDG-RQEKHP-IVLMWYF-C
5	STAND-G-RQEKHP-IVLMWYF-C
6	STAND-G-RQEK-HP-IVLMWYF-C
7	STA-ND-G-RQEK-HP-IVLMWYF-C
8	STA-ND-G-RQ-EK-HP-IVLMWYF-C
9	STA-ND-G-RQ-EK-HP-IVLM-WYF-C
10	ST-A-ND-G-RQ-EK-HP-IVLM-WYF-C
11	ST-A-ND-G-RQ-EK-H-P-IVLM-WYF-C
12	ST-A-N-D-G-RQ-EK-H-P-IVLM-WYF-C
13	ST-A-N-D-G-RQ-EK-H-P-IV-LM-WYF-C
14	S-T-A-N-D-G-RQ-EK-H-P-IV-LM-WYF-C
15	S-T-A-N-D-G-RQ-EK-H-P-IV-L-M-WYF-C
16	S-T-A-N-D-G-RQ-E-K-H-P-IV-L-M-WYF-C
17	S-T-A-N-D-G-RQ-E-K-H-P-IV-L-M-WY-F-C
18	S-T-A-N-D-G-R-Q-E-K-H-P-IV-L-M-WY-F-C
19	S-T-A-N-D-G-R-Q-E-K-H-P-I-V-L-M-WY-F-C

To further prove that polarity and hydrophobicity may be related to the function of 2OG oxygenases, we manually extracted the 2OG oxygenase domain and JmjC domain sequences and other iron-binding domain sequences for prediction. Protein functions mainly through its domain region, and 2OG oxygenases also bind Fe(II) and 2-oxoglutarate in their domain position to perform their functions. Therefore, the region outside the domain may be noise information for feature extraction, and only using the domain sequence to extract features can better reflect the function of 2OG oxygenases (Shen and Zou, 2020). The result is the same as we expected, when *t* = 33 and *s* = 15, the highest accuracy rate is obtained (Supplementary Figure 1B). The same result is obtained with the complete sequence, which further proves that the polarity and hydrophobicity may be related to the function of 2OG oxygenases.

The functional domain of 2OG oxygenases contains Fe^2+^-binding sites and α-ketoglutarate-binding sites, and their amino acid composition is almost completely conserved. The Fe^2+^-binding motif (HXD-H) and α-KG-binding motif (N-Y-R-R) of the ALKBH family are entirely conserved in the homologs (Bjornstad et al., 2011; Fedeles et al., 2015; Alemu et al., 2016; Xu et al., 2021), and other 2OG oxygenases have similar structures (Bleijlevens et al., 2008; Islam et al., 2018; Wang et al., 2021). They all combine Fe^2+^ and α-ketoglutarate through conserved polar amino acid regions, which may be the reason why polarity is an essential feature of 2OG oxygenase identification. In addition, in the best reduction scheme, Phenylalanine (F), Tryptophan (W), and Tyrosine (Y) are recombined into a new letter, and these three amino acids are all aromatic amino acids. We speculate that the function of 2OG oxygenases may be related to the hydrophobicity of aromatic amino acids and the unique properties of its benzene ring.

### Feature Selection

**FIGURE 5:**
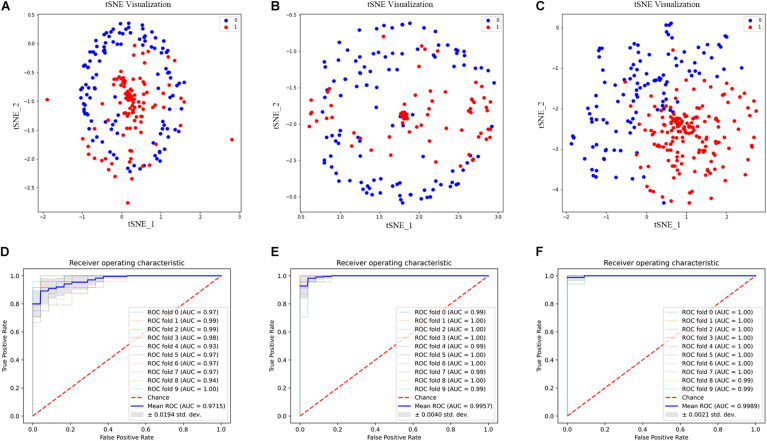
Feature set t-SNE clustering scatter diagram and receiver operating characteristic (ROC) curve diagram. **(A–C)** are the t-SNE clustering analysis diagrams of the feature set after unreduced, reduced, and feature screening, respectively. 0 and 1 represent positive samples and negative samples, respectively. **(D–F)** are the ROC curves of the three models 2OG-SwissProt, 2OG-Fe, and 2OG-domain, respectively.

Although we can get more features when *K* = 3, not every feature can be helpful to the prediction of 2OG oxygenases; some features may even become noise information and affect the final result. Therefore, we used ANOVA combined with IFS to select the best feature combination. Through 10-fold cross-validation, the 2OG-SwissProt model achieves an optimal performance of 91.46% with 812 feature combinations (Figure 4C); the 2OG-Fe model achieves an optimal performance of 96.61% with 1,181 feature combinations (Supplementary Figure 1C); 2OG-domain model also achieves an optimal performance of 96.07% with 350 feature combinations (Supplementary Figure 1C). For more clearly showing that the filtered features can better reflect the nature of 2OG oxygenases, we used t-Distributed Stochastic Neighbor Embedding (t-SNE) to visualize the feature sets after unreduced, reduced, and feature screening in a 2D feature space (Figures 5A–C). Obviously, the results show that the feature set clustering effect after feature screening is better, and it can effectively separate 2OG oxygenases from non-2OG oxygenases.

### Performance Evaluation

We evaluated our model by 10-fold cross-validation to verify that our model is effective (Table 3). At the same time, we drew the receiver operating characteristic (ROC) curve through the 10-fold cross-validation (Figures 5D–F).

**TABLE 3:** The results of each evaluation index of the three models.

Model	Acc (%)	Sn (%)	SP (%)	MCC (%)	*F*1score (%)	AUC (%)
2OG-SwissProt	91.04	93.33	88.75	82.34	91.26	97.15
2OG-Fe	97.23	97.92	96.53	94.48	97.31	99.57
2OG-domain	97.87	98.23	97.65	95.60	97.37	99.89

*Acc, accuracy; AUC, area under the curve; MCC, Matthew’s correlation coefficient; Sn, sensitivity; and Sp, specificity.*

In order to further evaluate our predictor, we used an independent test set to test 2OG-SwissProt, 2OG-Fe, and 2OG-domain models. The 2OG-SwissProt model accurately predicts 143 samples out of 150 test set samples, and the accuracy rate is 95.33%. The 2OG-Fe model accurately predicts 149 samples out of 159 test set samples, with an accuracy rate of 93.71%. The 2OG-domain model accurately predicts 963 samples out of 1,036 test set samples, with an accuracy rate of 92.95%. These show that our predictor is effective and robust.

### Web Server Guidance

**FIGURE 6:**
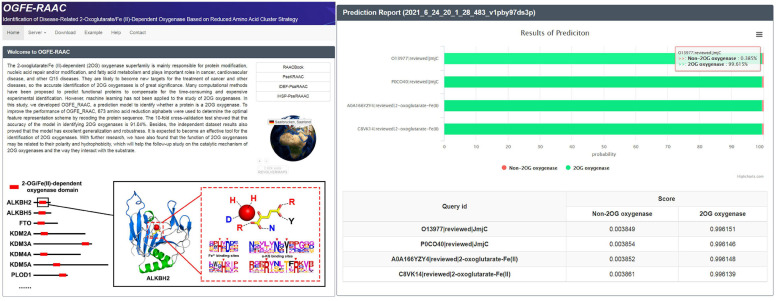
Home page and results page of OGFE-RAAC web server.

For the purpose of other researchers to use our model more conveniently, an easy-to-use web server was established to implement our predictor, which can be freely accessed at http://bioinfor.imu.edu.cn/ogferaac. When you want to use our tool, you need to click the “Service” module and then import the FASTA protein sequence into the input box or upload the button to upload your protein data. Meanwhile, according to the different sequences you provide, you can also choose different modules (2OG-SwissProt, 2OG-Fe, and 2OG-domain) for prediction. After submitting the task, the website will provide the corresponding forecast report, which will display the forecast results and probability of each sequence in the form of tables and flowcharts (Figure 6).

## Discussion

At present, the research on 2OG oxygenases is more in-depth, and its many functions (such as demethylation) occupy an important position in the research of diseases (Liu et al., 2019; Ao et al., 2021). Based on RAAC strategy and SVM, the prediction model of 2OG oxygenases is constructed. t-SNE results show that RAAC can effectively reduce protein complexity, extract conservative features hidden in noise information, and improve prediction accuracy. OGFE_RAAC has strong robustness and generalization to accurately predict 2OG oxygenases. We anticipate that OGFE_RAAC can accurately and rapidly identify 2OG oxygenases based on peptide sequence and promote the development of related drug research. Not only that, we also found that the function of 2OG oxygenases may be related to its hydrophobicity and polarity during the prediction process, which also provides a new research idea for the future study of 2OG oxygenases.

## Data Availability Statement

Publicly available datasets were analyzed in this study. This data can be found here: http://bioinfor.imu.edu.cn/ogferaac/public/Download.

## Author Contributions

YZ conceived and designed the study. JZ and PL organized and collected the data and carried out the computation. LZ designed and developed the web server. JZ and HW wrote the manuscript. SB participated in all subsequent revisions of the manuscript. YZ planned overall and revised the manuscript. All authors read and approved the manuscript.

## Conflict of Interest

The authors declare that the research was conducted in the absence of any commercial or financial relationships that could be construed as a potential conflict of interest.
