# Supplementary material for: Identification of Disease-Related 2-Oxoglutarate/Fe (II)-Dependent Oxygenase Based on Reduced Amino Acid Cluster Strategy
Source: Front Cell Dev Biol. 2021 Jul 16;9:707938. doi: 10.3389/fcell.2021.707938 (PMC8323781; doi:10.3389/fcell.2021.707938)
Supplement: Supplementary file 1 [file Image_1.pdf]

Supplementary Material

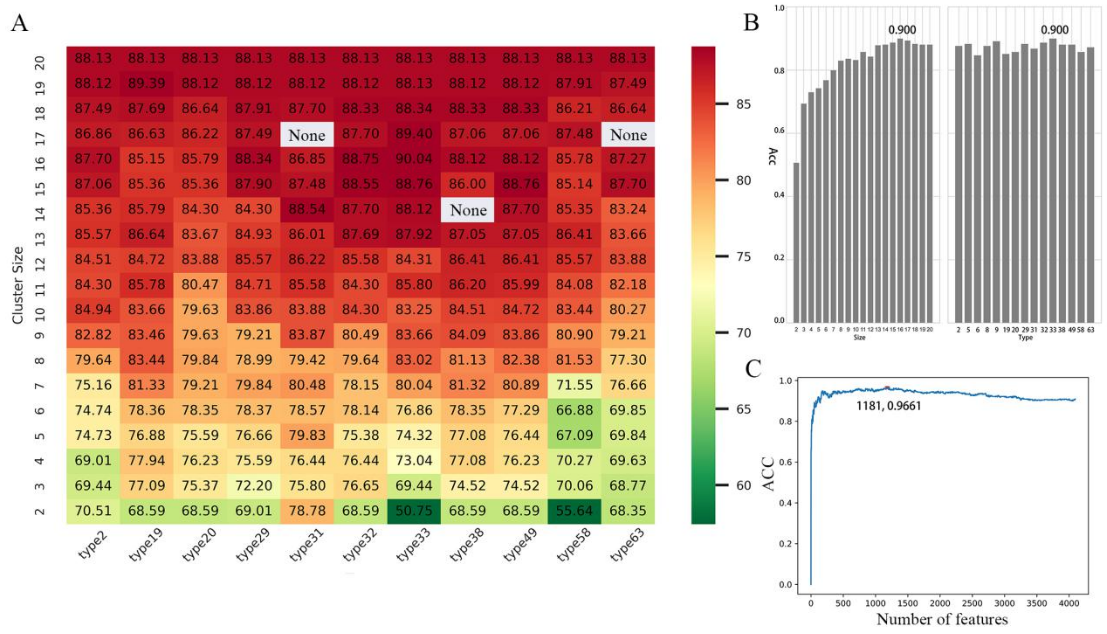

Supplementary Figure 1 | Performance evaluation of different reduced amino acid clusters. **(A)** Heat map of accuracy distribution of different reduced amino acid clusters. **(B)** The accuracy rate of the reduced amino acid cluster (t = 33, s = 16) with the highest accuracy rate reaches 90.04%. **(C)** The incremental feature selection (IFS) curve shows that prediction accuracy is 96.61% when uses 1181 optimal features based on the Tripeptide combination (t = 33, s = 16).

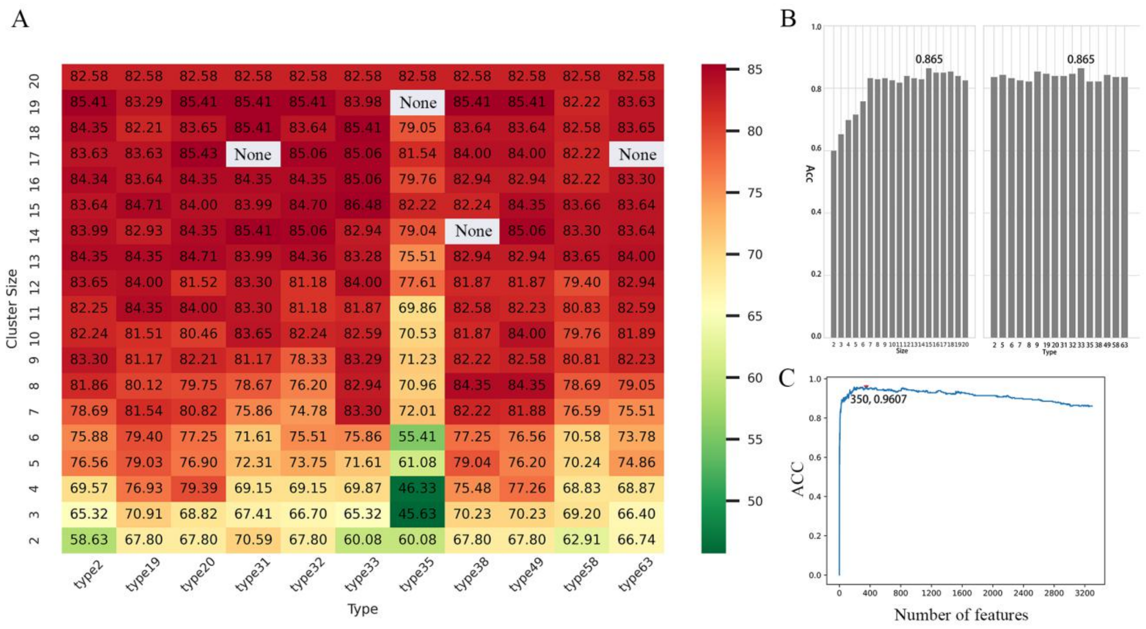

Supplementary Figure 2 | Performance evaluation of different reduced amino acid clusters. **(A)** Heat map of accuracy distribution of different reduced amino acid clusters. **(B)** The accuracy rate of the reduced amino acid cluster (t = 33, s = 15) with the highest accuracy rate reaches 86.48%. **(C)** The IFS curve shows that prediction accuracy is 96.07% when uses 350 optimal features based on the Tripeptide combination (t = 33, s = 15).
